# What Happens to the Immune Microenvironment After PD-1 Inhibitor Therapy?

**DOI:** 10.3389/fimmu.2021.773168

**Published:** 2021-12-23

**Authors:** Qingyi Wang, Bin Xie, Shuang Liu, Ying Shi, Yongguang Tao, Desheng Xiao, Wenxiang Wang

**Affiliations:** ^1^ Department of Pathology, Xiangya Hospital, Central South University, Changsha, China; ^2^ Department of Pathology, School of Basic Medicine, Key Laboratory of Carcinogenesis and Cancer Invasion (Ministry of Education), Central South University, Changsha, China; ^3^ National Health Commission (NHC) Key Laboratory of Carcinogenesis (Central South University), Cancer Research Institute and School of Basic Medicine, Central South University, Changsha, China; ^4^ Hunan Key Laboratory of Early Diagnosis and Precision Therapy, Second Xiangya Hospital, Central South University, Changsha, China; ^5^ Department of the 2nd Department of Thoracic Surgery, Hunan Cancer Hospital and The Affiliated Cancer Hospital of Xiangya School of Medicine, Central South University, Changsha, China

**Keywords:** immunotherapy, PD-1 inhibitor, tumor microenvironment, cytotoxic T lymphocytes (CTLs), immunotherapy resistance, combined immunotherapy

## Abstract

The fruitful results of tumor immunotherapy establish its indispensable status in the regulation of the tumorous immune context. It seems that the treatment of programmed cell death receptor 1 (PD-1) blockade is one of the most promising approaches for cancer control. The significant efficacy of PD-1 inhibitor therapy has been made in several cancer types, such as breast cancer, lung cancer, and multiple myeloma. Even so, the mechanisms of how anti-PD-1 therapy takes effect by impacting the immune microenvironment and how partial patients acquire the resistance to PD-1 blockade have yet to be studied. In this review, we discuss the cross talk between immune cells and how they promote PD-1 blockade efficacy. In addition, we also depict factors that may underlie tumor resistance to PD-1 blockade and feasible solutions in combination with it.

## Background

Immune surveillance functions of innate and adaptive immune cells can be suppressed by multiple mechanisms in the tumor microenvironment (TME); the most noted one is the programmed cell death receptor 1 (PD-1)/programmed cell death ligand 1 (PD-L1) pathway. For example, PD-L1, as the ligand of PD-1, could overexpress on tumor cells to evade the antitumor immune response by repressing the activation and function of CD8+T cells (1). Anti-PD-1 is one of the most promising attractive anticancer immune checkpoint blockers (ICB). Growing evidence shows that not only T cells but also other immune cells can be promoted by anti-PD-1 directly or indirectly, to suppress the progression of tumors (2–5). However, despite PD-1 blockade therapies having durable responses for a minority of patients in clinical trials, there is still an unmet clinical need for the majority of patients who do not respond to anti-PD-1 (6). Thus, we firstly summarize the cross talk between immune cells and their possible transformation in the TME after PD-1 blockade therapy. In the second part, we discuss the primary impact factors of resistance to PD-1 inhibitors, such as tumor immune recognition, oncogenic signal pathways, interferon (IFN), immune contexture, angiogenesis, immunometabolism, intestinal microbiota, and new immune checkpoints. We also highlight feasible combined therapy strategies to re-sensitize tumors to PD-1 blockade.

## The Role of PD-1 and PD-1 Inhibitors in Immune Response

### PD-1

PD-1, a member of the B7-CD28 receptor family, is a transmembrane protein and widely expressed in B cells, T cells, natural killer (NK) cells, and myeloid cells (7). As the ligand of PD-1, programmed cell death ligand 1 (PD-L1) can be expressed in dendritic cells (DCs), macrophages, T cells, NK cells (8, 9), and tumor cells (10). Generally, when PD-L1 binds to PD-1 in the presence of the T cell receptor (TCR) signaling complex, PD-1 delivers a co-inhibitory signal, leading to the termination of TCR signaling and inhibition of T cell proliferation (11). PD-1 often uses mono-tyrosine signaling motifs which present in its cytoplasmic tail, such as immunoreceptor tyrosine-based inhibitory motif (ITIM) and immunoreceptor tyrosine-based switch motif (ITSM) (12), to end the CD28/TCR signal by PD-1 phosphorylation and the recruitment of SHP-2 and SHP-1 (13–15). In the tumor immune context, antigen-presenting cells (APCs) and tumor cells highly express PD-L1, and they can interact with PD-1-overexpressed T cells, leading to T-cell anergy or exhaustion (16, 17). Programmed cell death ligand 2 (PD-L2) is the second ligand for the PD-1 molecule, which is expressed predominantly by DCs, macrophages, B cells, and cancer cell populations, depending on microenvironmental stimulation (18, 19). Similar to PD-L1, PD-L2 plays a crucial role in evading antitumor immunity. The engagement of PD-1 and PD-L2 can lead to the downregulation of T cell responses, which inhibits TCR-mediated proliferation and cytokine production by CD4+ T cells by blocking cell cycle progression (18). Although PD-1/PD-L2 blockade must be considered for optimal immunotherapy in antitumor immunity (20), since most of the research results are focused on the PD-1/PD-L1 pathway, we mainly discuss the PD-1/PD-L1 axis in this article.

### PD-1 Inhibitors

As surface molecules, the activity of PD-1 and PD-L1 can be easily inhibited by blocking antibodies. Anti-PD-1 therapy is one of the most successful immune checkpoint blockade therapies that have been approved to treat a wide variety of cancer types (**Table 1**). PD-1 inhibitors competitively bind to PD-1 and block PD-1/PD-L1 interactions, which subsequently regulate negative signals on the T cell surface to enhance the functions of effector T cells and promote the proliferation of T cells (54). Nivolumab and pembrolizumab are the primary clinically approved PD-1 inhibitors. They are humanized IgG4 antibodies targeting PD-1 with high affinity (55). To ensure that they elicit their inhibitory effects of PD-1/PD-L1 interactions primarily by direct occupancy and steric blockade of the PD-L1-binding site of PD-1 (56), they minimize the function of effector cells engaging other antibodies.

**Table 1:** Summary of FDA-approved PD-1 inhibitors in advanced/metastatic cancers.

Agent(s)	Pathology	Indications	Clinical trial	Reference
Pembrolizumab	Melanoma	First-line/ Second-line	KEYNOTE-006 phase 3/KEYNOTE-002 phase 2	(21, 22)
	NSCLC	First-line (TPS ≥1%, ALK/EGFR wt) Second-line (TPS ≥1%)	KEYNOTE-042 phase 3/KEYNOTE-010 phase 2/3	(23, 24)
	HL	Relapsed after ≥ third-line	KEYNOTE-087 phase 2	(25)
	PMBCL	Relapsed after ≥ second-line	KEYNOTE-170 phase 2	(26)
	MCC	First-line	KEYNOTE-017 phase 2	(27)
	UC	First-line cisplatin-ineligible/recurrent after platinum-based treatment	KEYNOTE-052 phase 2/KEYNOTE-045 phase 3	(28, 29)
	HCC	Second-line after sorafenib	KEYNOTE-224 phase 2	(30)
	GC	Progression on or after ≥ second-line (CPS ≥1%)	KEYNOTE-059 phase 2	(31)
	Non-colorectal MSI-H/dMMR cancer	Previously treated	KEYNOTE-158 phase 2	(32)
	HNSCC	First-line (CPS ≥1%)/ Second-line	KEYNOTE-048 phase 3 KEYNOTE-012 phase 1b	(33)
	CC	Previously treated (CPS ≥1%)	KEYNOTE-158 phase 2	(34)
	EC	Progression after first-line (CPS ≥10%)	KEYNOTE-181 phase 3	(35)
Pembrolizumab + chemotherapy	NSCLC	First-line	KEYNOTE-021 phase 2/KEYNOTE-407 phase 3	(36, 37)
	EC	First-line	KEYNOTE-590 phase 3	(38)
Nivolumab	Melanoma	First-line/second-line	CheckMate-037/066 phase 3	(39, 40)
	NSCLC	Second-line	CheckMate-017/057 phase 3	(41, 42)
	HL	Progressed after ASCT or brentuximab	CheckMate-039 phase 1 CheckMate-205 phase 2	(43)
	UC	Recurrent after platinum-based treatment	CheckMate-275 phase 2	(44)
	HCC	Previously treated with sorafenib	CheckMate-040 phase 1/2	(45)
	MSI-H/dMMR colorectal cancer	Treatment-refractory to all standard therapies	CheckMate-142 phase 2	(46)
	HNSCC	Platinum-refractory, recurrent	CheckMate-141 phase 3	(47)
	SCLC	Third-line	CheckMate-032 phase 1/2	(48)
Nivolumab+ Ipilimumab	MSI-H and dMMR	Treatment-refractory to all standard therapies	CheckMate-142 phase 2	(49)
	RCC	First-line	CheckMate-214 phase 3	(50)
	NSCLC	First-line (PD-L1 ≥1%)	CheckMate-227 phase 3	(51)
Cemiplimab	CSCC	First-line	NCT02383212/ NCT02760498 phase 3	(52)
	NSCLC	First-line (TPS ≥50% EGFR, ALK, or ROS1 wt)	NCT03088540 phase 3	(53)

Tumor types: NSCLC, non-small cell lung carcinoma; HL, Hodgkin lymphoma; PMLBCL, primary mediastinal large B cell lymphoma; MCC, Merkel cell carcinoma; UC, urothelial carcinoma; HCC, hepatocellular carcinoma; GC, gastric cancer; MSI, microsatellite instability; dMMR, mismatch repair-deficient; HNSCC, head and neck squamous cell carcinoma; CC, cervical cancer; EC, esophageal cancer; SCLC, small cell lung carcinoma; RCC, renal cell carcinoma; CSCC, cutaneous squamous cell carcinoma. wt, wild-type; TPS, tumor proportion score; CPS, combined positive score; ASCT, autologous hematopoietic stem cell transplantation.

Pembrolizumab was initially approved for refractory unresectable melanoma in 2014 (57), known as the first PD-1-targeted therapy to gain Food and Drug Administration (FDA) approval. Not long, in 2015, it becomes the first immune checkpoint inhibitor to be approved as a first-line treatment, also in melanoma therapy (21). Pembrolizumab is thus approved to treat a wide variety of cancer types. To date, pembrolizumab therapy has been licensed in many cancers (27, 30, 58, 59) and was often conducted primarily in patients with PD-L1-positive disease (31, 34). In general, a higher level of PD-L1 expression is associated with a more effective clinic outcome of pembrolizumab. However, in some cancer types, such as non-small cell lung cancer (NSCLC) (60), classical Hodgkin’s lymphoma (cHL) (25), and urothelial carcinoma (UC) (61), PD-L1 expression did not explicitly correlate with response to pembrolizumab.

Nivolumab also displays a good response and favorable safety profile, particularly in melanoma and NSCLC. Nivolumab was approved by FDA following its showing a clear advantage in response over chemotherapy in refractory unresectable melanoma (62). Soon after, the FDA approved nivolumab for the treatment of NSCLC after progression on a platinum-based chemotherapy regimen (41, 63). Also, nivolumab has been demonstrated durable effects in other cancers (47, 64, 65), and it appears that combination therapy may further improve them (50, 66). Nevertheless, research has demonstrated a low response rate in some hematological tumors, such as follicular lymphoma (FL) (67) and diffuse large B cell lymphoma (DLBCL) (68). It may appear to correlate positively with 9p24.1 translocation and increased PD-L1 expression (69). In addition to nivolumab and pembrolizumab, cemiplimab is also approved by FDA for the treatment of advanced cutaneous squamous cell carcinoma (70) and first-line NSCLC (53). Up till now, more than 1,500 clinical trials involving PD-1 inhibition are currently supported by the National Cancer Institute (NCI).

## Immune Microenvironment

**Figure 1:**
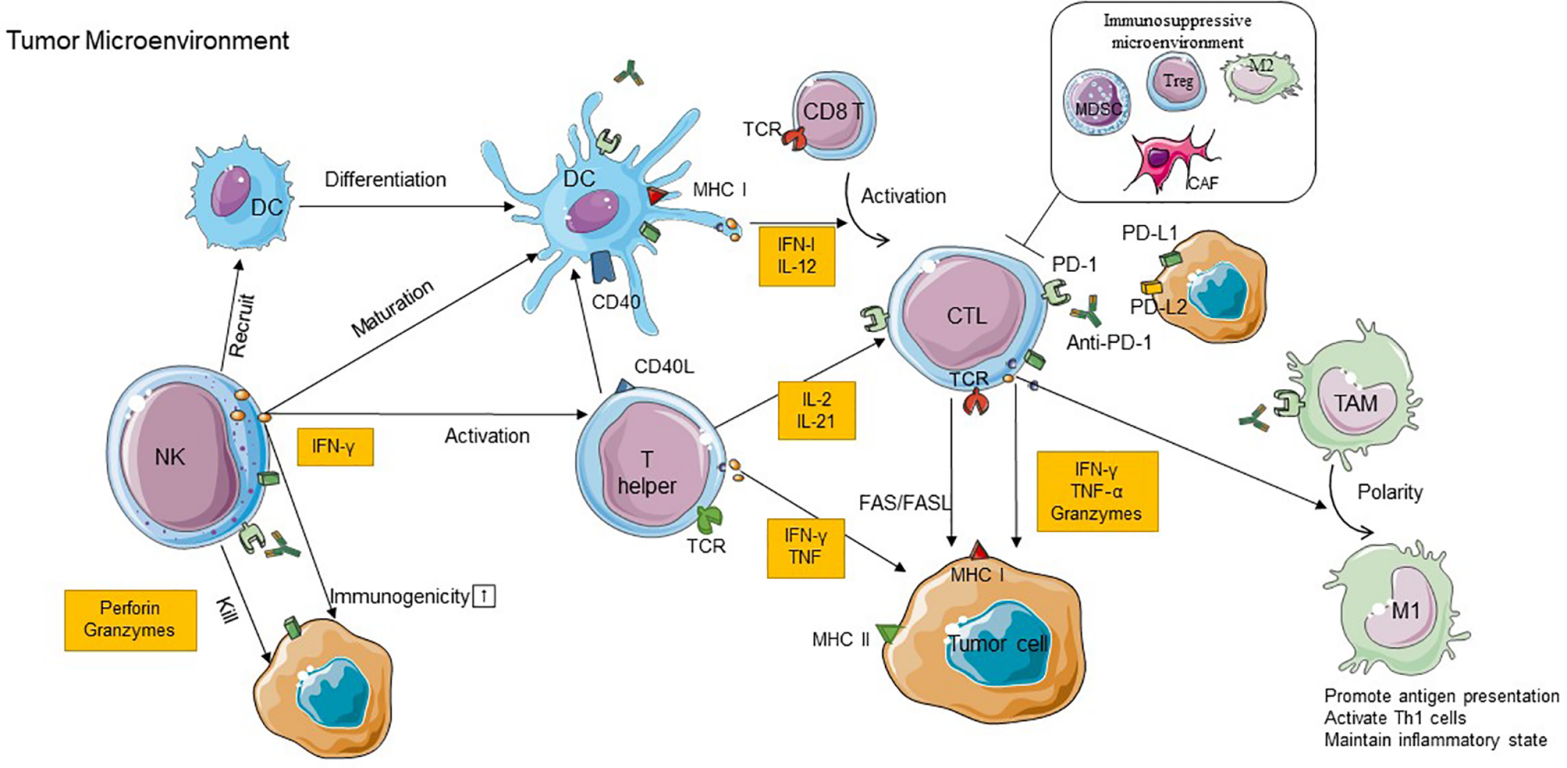
The cross talk between immune cells in TME and the role of immune cells in the efficacy of PD-1 inhibitor therapy. CD8+T cells recognize antigens requiring a corroboration work between CD4+T cells with NK cells and DCs, and M1 TAM can exert antitumoral effects due to the stimulation of IFN-γ produced by CD8+ T cells. In addition to these immunostimulatory cells, the immunoinhibitory cells including CAFs, Tregs, M2 TAMs, and MDSCs can construct an immunosuppressive microenvironment to restrict the antitumor effect. Anti-PD-1 binds to PD-1 on immune cells which can block PD-1/PD-L1 interactions and recover the antitumor function of those cells. PD-1, programmed cell death protein 1; PD-L1, programmed death-ligand 1; PD-L2, programmed death-ligand 2; IFN-γ, interferon gamma; TNF, tumor necrosis factor; CTL, cytotoxic T lymphocyte; DC, dendritic cell; NK, natural killer cell; MDSC, myeloid-derived suppressor cell; Treg, regulatory T cell; CAF, cancer-associated fibroblast; TAM, tumor-associated macrophage; M1, type 1 macrophage cell; M2, type 2 macrophage cell; MHC, major histocompatibility complex; TCR, T cell receptor; TME, tumor microenvironment.

Immunotherapies based on PD-L1/PD-1 blockade have revolutionized the treatment paradigm for several cancer types. Their interaction regulates the activation of immune responses and specifically of T cell responses in physiological conditions. In the last years, increasing evidence has demonstrated that the elimination of tumor cells is mainly mediated by cytotoxic T lymphocytes (CTLs) (71). Several types of immune cells in the TME, such as tumor-associated macrophages (TAMs), DCs, NK cells, and immunosuppressive cells, can also interact with each other to promote or repress tumor progression in direct and indirect mechanisms by secreting cytokines and chemokines (71). Indeed, there is a complex picture of the relationship between checkpoint blockade and immune context. The precise molecular mechanisms of how PD-1 inhibitors function by stimulation/inhibition of immune-related cells remain to be fully understood. Here, we will attempt to discuss in detail the cross talk between immune cells and the critical role of some immune cells in the efficacy of PD-1 inhibitors therapy (**Figure 1**).

### T Cells

#### CD8+T Cells

CD8+T cells are a subset of lymphocytes developing in the thymus. They recognize antigen-presented cells expressing major histocompatibility complex (MHC) class I molecules and in turn exert antitumor function (3, 71). Initiation of a response from CD8+T cells against an antigen requires corroboration work between CD4+T cells with NK cells and DCs (3, 72). Activated, antigen-loaded DCs can launch the differentiation of CD8+T cells into CTLs by cross-presenting MHC class I molecules to cells (73). CD4+T cells can secrete cytokines following the interaction with antigens to simulate the optimal proliferation and activation of CD8+ T cells (74). On the other hand, NK cells and CD4+T cells can produce chemokines which indirectly induce the activation of CD8+ T cells by promoting the differentiation and maturation of DC cells (72, 75). Due to such cross talk, CTLs can initiate the antitumor effect through releasing IFN-γ and tumor necrosis factor α (TNF-α) to induce cytotoxicity in the cancer cells (76).

However, PD-1, as a coinhibitory receptor, could overexpress on activated CD8+ T cells (77). Once this happens, signals downstream of TCR may be attenuated and may cause the exhaustion of CD8+ T cells and ultimately contribute to the restriction of T cell activation and cytokine production (78). PD-1 blockade therapy seems to counteract tumor-induced T cell dysfunctionality by interfering with PD-1/PD-L1 signals; it releases the negative regulation of T cells and promotes T cells which produced higher levels of IFN-γ to activate antitumor immune response (79–81). Besides, PD-1 inhibitors reinvigorate preexisting CD8+T cells within the tumor and promote systemic T cell immunity priming. Nevertheless, the study revealed that preexisting tumor-specific T cells may have limited reinvigoration capacity and that the T cell response to checkpoint blockade derives from a distinct repertoire of T cell clones that may have just recently entered the tumor (82). The priming of antitumor T-cell immunity in lymphatic drainage might explain such consequence, which is further explained in another study. This study showed that tumor-draining lymph nodes (TDLNs) are enriched for tumor-specific PD-1^+^T cells which are closely associated with PD-L1^+^DCs (83). Suppression of DCs, accompanied by excess PD-L1 surface expression, may lead to restrained T cell priming and deviated CD8+ T cell differentiation in the TDLN. Therefore, it suggests that progenitor-exhausted T cells can be rescued by immune checkpoint blockade and then home to the tumor and populate the TME, to improve tumor control (83). However, the exact contribution of TDLN versus TME during PD-1/PD-L1 checkpoint blockade therapy remains to be elucidated.

On the other hand, the report found that PD-L1 can also be upregulated on T cells (84). PD-L1-expressing T cells can suppress immunity on neighboring T cells and polarize macrophages toward a tolerogenic phenotype *via* the PD-L1–PD-1 axis in the TME, which in turn both suppresses T cell activation and promotes tumor growth (84). It is still not clear whether PD-1 inhibitors also play a role based on this theory. Accordingly, the precise molecular mechanisms of T cell function stimulated by PD-1 inhibitors remain to be clarified.

#### CD4+T Cells (T Helper Cells)

CD4+T cells participate in the activation and expansion of CD8+T effectors; they induce an antitumor response by providing regulatory signals (85–87). In the tumor context, MHC class II molecules can present antigenic peptides recognized by CD4+T cells (88, 89). MHC-class II+ tumors can be directly killed by CD4+ CTLs. For the MHC-class II-negative tumor cells, CD4+ T cells can produce a vast range of cytokines that mediate inflammatory and effector immune responses (90, 91); TNF and IFN-γ are the most important cytokines that are mainly produced by T helper (Th) 1 cells. Additionally, CD4 Th1 cells also display antitumor responses by activating NK cells (90) and M1 TAM (92, 93), inhibition of angiogenesis (94), and/or induction of tumor senescence (95).

To date, the specific contribution of CD4 immunity to PD-1 blockade therapy efficacy is still unknown. In NSCLC, proliferation and low PD-1/LAG-3 co-expression of CD4 at baseline were responsive to PD-1 blockade *ex vivo* and *in vivo* (96). In cHL, PD-1 blockade therapy has strong antitumor effects on MHC-II-expressing tumors mediated by cytotoxic CD4+ T cells in murine models (97). These provide strong evidence that CD4 immunity might be an entry point to achieve efficacious clinical responses under PD-1 blockade therapies. Further research is needed to reveal the specific contribution of CD4+ T cells.

### NK Cells

NK cells can spontaneously kill cells and thus are presumed to be key innate immune effectors in cancer immunosurveillance; it belongs to the family of innate lymphoid cells (ILCs) (98). IFN-γ produced by NK cells during early-phase immune responses can directly kill tumor cells and promote the differentiation of naive CD4+ T cells toward Th1 cells to facilitate cell-mediated immunity (99). Thus, NK cells are critical components both in humoral immunity and in cellular immunity.

As an inhibitory receptor, PD-1 can express on NK cells (100, 101) and prevent the activation of NK cell function when engaging with its ligand which is expressed on the surface of target tumor cells or APC (102). PD-1^+^ NK cells may be inhibited in killing tumor cells instead of being anergic in PD-L1^+^ tumors, which means that PD-1 is an important checkpoint for NK activation and PD-1 blockade might elicit an antitumor NK cell response (102). In high PD-L1 expression head and neck cancer (HNC) patients, the study observed that PD-1 blockade increased cetuximab-mediated NK cell activation and cytotoxicity (103). Besides, tumors might drive the development of PD-L1-expressing NK cells that acquire immunoregulatory functions; such cell population can directly inhibit CD8+ T cell proliferation in a PD-L1-dependent manner (104). These results show the importance of the PD-1/PD-L1 axis in inhibiting NK cell responses *in vivo*, and future research is needed to determine the specific mechanism of the PD-1 pathway in the antitumor response of NK cells.

### DCs

DCs, known as specialized APC, transport tumor antigens to draining lymph nodes and cross-present antigens *via* MHC I and II to activate cytotoxic T lymphocytes (105). DC maturation is necessary to T cell proliferation and differentiation; the final antitumor immunity is also associated with co-stimulatory molecules and cytokines which are expressed as the mature markers on DCs, such as CD80/CD86 and IL-12 (106).

DCs are necessary for anti-PD-1 efficacy. Anti-PD-1-activated T cells secrete IFN-γ, which in turn primes a transcriptomic shift in DC phenotype; DCs produce IL-12 upon sensing IFN-γ to stimulate effector T cell responses (107–109). The activation of the non-canonical nuclear factor kappa-light-chain enhancer of the activated B cell (NF-κB) pathway is also required for checkpoint efficacy, for it can enrich IL-12-producing DCs (107). Additionally, evidence of direct regulation is still emerging. PD-1 expression has recently been identified on DCs in the specific tumor context (110, 111). The result of an ovarian study demonstrated that PD-1 expressed on the tumor-associated DC can suppress NF-κB activation and the release of immune regulatory cytokines and restrict the upregulation of co-stimulatory molecules (111), which mediate immune suppression. PD-1 inhibition seems to increase the co-stimulatory molecule expression of DCs (112). In addition, the specific ablation of PD-1 on intratumoral DCs resulted in enhanced priming of tumor-specific CD8+ T cells to secrete IL-2 and IFN-γ (110). While DCs are the major antigen-presenting cells for cross-presenting tumor antigens to T cells and promoting antitumor response, PD-L1 expression on DCs can be upregulated by inflammatory cytokines, especially IFNs. Such upregulation is likely to prevent the overexpansion of tumor-infiltrating lymphocytes and eventually dampen the antitumor responses (113, 114). These results might provide additional insights into the role PD-1/PD-L1 plays on DCs to facilitate antitumor response and the mechanisms of immune checkpoint blockade therapy efficacy.

## TAMs

TAMs are major components of infiltrated leukocytes in tumors, which dominantly orchestrate cancer-related inflammation (115). They can be divided into two subtypes: M1 and M2. Anti-tumorigenic M1 macrophages express high levels of TNFα, inducible nitric oxide synthase (iNOS), and MHC class II molecules. They exert antitumoral effects due to the stimulation of IFN-γ produced by CD8+ T cells and CD4+T cells (71). Inversely, pro-tumorigenic M2 macrophages are marked with a high level of arginase 1 (ARG1) and CD206 expression (116). M2 cells can secrete STAT3 to the TME for impairing responses from CTLs when their number increases in the stroma (117). Besides, M2 cells can express inhibitory ligands PD-L1, which bind to inhibitory receptor PD-1 constitutively expressed in T cells to activate them, directly inhibiting TCR signals to restrain the antitumor function of T cells (118).

Primary macrophages transform into the M1 or M2 phenotype which can be induced by PD-1 signaling pathways (119). TAMs display detectable PD-1 levels in the tumor microenvironment; PD-1 blockade therapy contributed to both a direct and an indirect impact on TAMs. Indirectly, checkpoint blockade-activated T cells can accumulate TAMs by secrete factors (such as IFN-γ) to remodel the TME toward a tumor hostile environment rich in iNOS+ TAMs (119). In direct regulation, PD-1 deficiency in TAMs shifts their phenotype toward an antitumor profile, with higher levels of TNF-α, iNOS, and MHC II (120). Myeloid-specific PD-1 deletion was as effective at limiting tumor growth as global PD-1 deletion and more effective than selective ablation of PD-1 in T cells (121). TAM PD-1 expression negatively correlates with phagocytic potency against tumor cells; TAM infiltration is skewed toward high CD206 and ARG1 macrophages dampening antitumor immune responses (122, 123). Anti-PD-1 therapy can surprisingly reverse this trend, increasing the expression of iNOS, TNF-α, and IL-6, which may augment antitumor immunity (124). Accordingly, the inhibition of PD-1 expressed on TAMs can shift them to the M1 phenotype and form an antitumor TME.

### Immunosuppressive Cells

Immunosuppressive cells, unlike immune cells, have a positive effect on antitumoral immunity. There are some immunoinhibitory cells that display negative cross talking in TME, including cancer-associated fibroblasts (CAFs), regulatory T cells (Tregs), myeloid-derived suppressor cells (MDSCs), and M2 TAMs (mentioned above). Tregs repress the proliferation of both CD8+ and CD4+T cells through releasing transforming growth factor β (TGF-β) (125). CAFs promote the rate of glycolytic metabolism and further constitute a glucose-deficient TME. CTLs tend to decrease their number when encountered with such conditions (126). It is not yet known whether these have a role in promoting the efficacy of PD-1, but studies have shown that they are crucial in immune resistance, which will be discussed in detail in a subsequent paragraph.

## Drug Resistance and Combined Therapy

**Figure 2:**
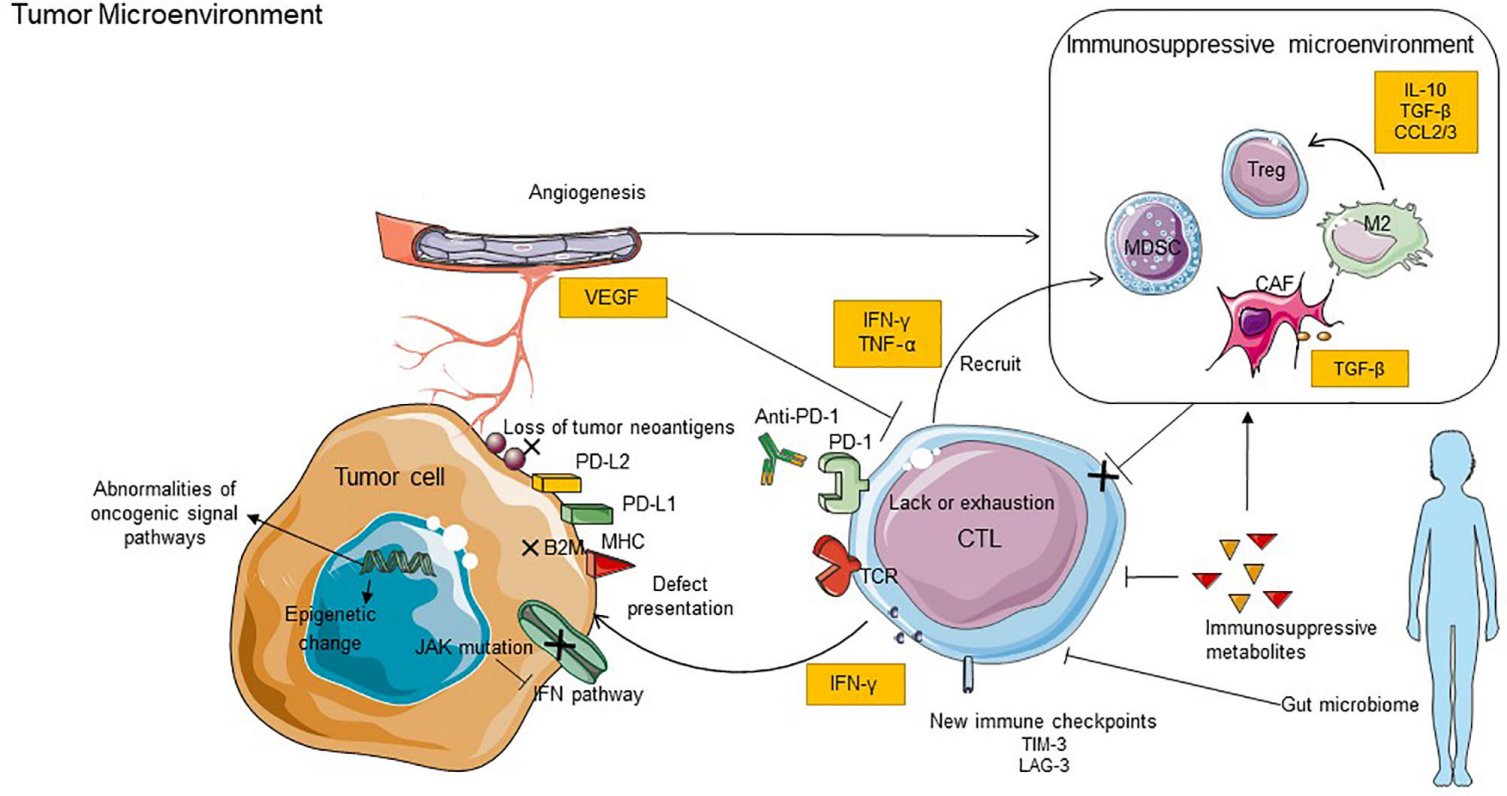
Key mechanisms of resistance to anti-PD-1 inhibitors. The mechanisms of resistance including internal and external causes. The internal causes focus on the inherent characteristics of tumor cells, it includes defective tumor immunorecognition, epigenetic regulation, abnormal oncogenic signaling, and IFN-γ signal pathway. The external causes are mainly emanated from the tumor microenvironment, such as exhaustion of T cells, immunosuppressive cells and cytokines, tumor metabolites, new immune checkpoints, and intestinal microflora. B2M, B2-microglobulin; LAG3, lymphocyte-activation gene 3; TIM3, T cell immunoglobulin and mucin domain-containing molecule 3; VEGF, vascular endothelial growth factor; JAK, Janus kinase.

Anti-PD-1 therapy has shown significant efficacy in clinical trials and has been approved for treating several cancers in clinic therapy. However, the occurrence of primary or acquired drug resistance will cause the patient to be ineffective to PD-1 blockade therapy or eventually the recurrence of malignant tumors (127). There are internal and external causes of tumor resistance to PD-1 blockade. The internal causes focus on the inherent characteristics of tumor cells; these include defective tumor immunorecognition, epigenetic regulation, abnormal oncogenic signaling, and IFN-γ signal pathway, while the external causes are mainly emanated from the tumor microenvironment, such as exhaustion of T cells, immunosuppressive cells and cytokines, tumor metabolites, new immune checkpoints, and intestinal microflora (128). Here, we summarize the primary resistant mechanisms to anti-PD-1 (**Figure 2**). In addition, we highlight emerging combined treatment strategies that might prolong the efficacy of PD-1 blockade or enable immunotherapy to impinge on previously intractable cancer types.

### Defective Tumor Immunorecognition

Some studies have shown that carcinomas with robust T cell immunosurveillance can evade recognition through diverse genetic and immune-related mechanisms, including loss of tumor neoantigens and defect in antigen presentation.

#### Loss of Tumor Neoantigens

Despite that cancer immunoediting can suppress tumor growth, it can establish favorable conditions within the tumor microenvironment to facilitate tumor outgrowth of the immune system which no longer recognizes the tumor (129). A neoantigen is an antigen encoded by the mutant gene of tumor cells. It is cross-presented *via* DCs and recognized by mature activated T cells. Emerging research supports the critical role of neoantigens in response to PD-1 blockade therapy. For instance, it highlights that neoantigen-specific CD8+T-cell responses were parallel to tumor regression in a responder of NSCLC patients treated with pembrolizumab (130), indicating that anti-PD-1 therapy enhances tumor neoantigen-specific T cell responses. In addition, in NSCLC patients who developed acquired drug resistance after single anti-PD-1 or anti-PD-1 combined with anti-CTLA-4 therapy, the loss of neoantigens has been found based on complete exome sequencing of tumor cells (131). It means that the PD-1-blocking therapy may be less effective if the tumor does not contain a mutation that can be a target. Despite the underlying mechanism being still unclear, evidence highlights that the combination of radiotherapy (RT) and anti-PD-1 is considered a promising strategy (132). Most likely, it is dependent on RT-induced cell damage that may express somatic mutations that generate neo-antigens, which have the potential to serve as targets for a more robust immune response (133). In preclinical triple-negative breast tumor models, data show that radiotherapy can enrich tumors of functionally active. Curative capacity has been enhanced when radiotherapy is combined with immunostimulatory and α-PD-1 monoclonal antibodies (mAbs) (134). Similarly, cancer cell death induced by chemotherapy is thought to promote tumor antigen release and antigen presentation and stimulate immune effectors. Combining checkpoint inhibitors with standard-of-care chemotherapy has been successful in non-small cell lung carcinoma (135, 136) and triple-negative breast cancer (137). Besides, individualized mutanome vaccines, an RNA-based poly-neo-epitope approach to mobilize immunity against a spectrum of cancer mutations, were applied to patients in melanoma and obtained a complete response to vaccination in combination with PD-1 blockade therapy (138). These results mean that the combination of PD-1 blockade with an agent that can facilitate tumor cells to generate neo-antigen may increase antitumor immunity.

#### Defective Antigen Presentation

Effective tumor antigen presentation to CD8+T cells relies on class I MHC (139, 140). Loss of heterozygosity and genetic deficiencies of β2-microglobulin (B2M) are both crucial ways that lead to the loss of MHC molecules (140–142), which promote resistance to PD-1 blockade due to the inability of CD8+T cells to recognize tumor antigens and specifically kill tumor cells (143). Thus, to recover the ability of antigen presentation may represent potential avenues that can be combined with immunotherapy.

The impairment of antigen presentation can be induced by epigenetic regulation. DNA methylation is thought to regulate the expression of tumor-associated antigens by downregulating the level of MHC class I. Studies have shown that the capability of DNA methyltransferase inhibitors (DNMTi) to upregulate MHC class I and MHC class II has appeared in many cancers (144, 145). Enhancer of zeste homolog 2 (EZH2), a catalytic component in the polycomb repressive complex 2 (PRC2), plays a crucial role in the mediation of histone h3 lysine 27 tri-methylation (H3K27me3) (146). Research revealed a negative correlation between the expression levels of EZH2 and MHC I antigen presentation molecules (147). The study also found that tumor progression of an anti-PD-1-resistant head and neck squamous cell carcinoma (HNSCC) model can be suppressed by combinatorial treatment of an EZH2 inhibitor and anti-PD-1. Paradoxically, in ovarian cancer models, EZH2 inhibition has nothing to do with the alteration of the class I antigen presentation of ovarian cancer cells (148), indicating that the regulation of EZH2 on antigen presentation may be cancer-type specific. Therefore, the impairment of antigen presentation may promote tumor immune escape while providing a potential strategy to overcome resistance to PD-1 inhibitor therapy.

### Oncogenic Signal Pathways

Cancer is a genetic disease that can be induced by multiple genetic alterations, which are commonly caused by abnormalities of several key oncogenic pathways (149), like the phosphatase and tensin homolog (PTEN) signal pathway and mitogen-activated protein kinase (MAPK) signal pathway. Here, we mainly describe the two most common pathways, which have been proven to be closely related to PD-1 inhibitor resistance.

Research found that loss of PTEN in tumor cells in clinical patients of melanoma correlates with decreased T-cell infiltration, expansion, and inferior outcomes with PD-1 inhibitor therapy (150). PTEN loss-of-function mutations in tumors were significantly increased in non-responders who were treated with anti-PD-1 antibodies (151). Additionally, one of the most common pathways activated by loss of expression of the tumor suppressor PTEN is the phosphatidylinositol 3-kinase (PI3K) pathway, which plays a critical role in cancer by regulating several critical cellular processes. Thus, the PI3Kβ inhibitor, which is thought to regulate AKT activity in tumors with PTEN loss, has been applied to PTEN-deficient melanoma mouse models and demonstrated to enhance the efficacy of both PD-1 and CTLA-4 inhibitors (150). Accordingly, the regime that anti-PD-1 combined with PI3K-AKT pathway inhibitors may benefit cancer patients in the future.

The RAF/MEK/ERK pathway which is the classic routine in the MAPK pathway is also critical for human cancer; the pathway can be primed by activated RAS interacting with RAF kinase (152–154). Furthermore, RAS, RAF, and MEK are also frequently amplified or mutated in various cancers, accompanied by the activated MEK-ERK signaling pathway (155). KRAS, the component of RAS, is one of the most frequently mutated oncogenes in human cancers and participates in the mechanism of PD-1 inhibitor resistance (156). Similarly, BRAF, another mutated oncogene, has the vast majority in number harboring an activating point mutation (V600E) (157). This oncogenic mutation leads to constitutive activation of the MAPK signaling pathway and increased oncogenic potential through a variety of mechanisms, including reduced apoptosis, increased invasiveness, and increased metastatic behavior (158). Recent *in vitro* data suggest that BRAF V600E could also contribute to immune escape (157, 159). Based on these, selective inhibition of BRAF has been shown to induce an activated CD8+ T cell infiltrate, as well as increase melanoma MHC expression and melanoma antigen presentation early during treatment both in preclinical models and in human melanoma tissue samples (159–161). The study also suggested that combined BRAF and MEK inhibition with PD-1 blockade immunotherapy in BRAF-mutant melanoma can increase the frequency of long-lasting antitumor responses (162). Thus, the inhibition of the RAF/MEK/ERK signaling pathway may be a promising therapeutic strategy for cancer dysregulated in this pathway.

### IFNs

IFN-γ, effector cytokines of T cells, can directly exert an effective antitumor immune response by recognizing the corresponding receptors on tumor cells or indirectly promote the cross-activation of CD8+ T cells by upregulating antigen-presenting machinery to attack tumor cells (163). Classically, IFN-γ inhibits the proliferation of tumor cells and promotes their apoptosis, as it can activate signal transducer and activator of transcription 1 (STAT1) through using the Janus kinase (JAK) signal transducer and activator of the transcription pathway (127). Recent studies have implicated that defects in such pathways involved in IFN-receptor signaling and antigen presentation are associated with primary and acquired resistance to PD-1 blockades, such as inactivating mutations in JAK1 and JAK2 (143, 164). It may result in PD-L1 not being able to be reactively expressed and failing to attract T cell infiltration due to lack of chemokine production which is controlled by the IFN-γ pathway downstream of JAK1/2 (165). Considering that preexisting T cells in the tumor are a requisite for response to anti-PD-1 therapy (166), the absence of reactive PD-L1 expression may implicate a poor response to PD-1 blockade therapy, because of the impairment of tumor-infiltrating T cells (164).

IFN-β, belonging to type I IFN that is associated with innate immune responses (167), was proved to be suppressed by lysine-specific histone demethylase 1 (LSD1) (168). Ablation of LSD1 in cancer cells increases repetitive element expression; this leads to dsRNA stress and activation of type 1 IFN, which promotes antitumor T cell immunity and sensitizes refractory tumors to PD-1 blockade in a melanoma mouse model (168). The remarkable ability of LSD1 inhibition to convert a tumor resistant to PD-1 blockade to a tumor responsive to PD-1 blockade provides a means to increase the efficacy of anti-PD-1 cancer therapy and potentially turn “cold” tumors “hot” (169). It may suggest LSD1 inhibition combined with PD-1 blockade as a novel cancer treatment strategy. In addition, long-term IFN-β transcription can also promote the occurrence of resistance to anti-PD-1 therapy by inducing intratumoral augment of Tregs and myeloid cells, which cause T cell depletion and immunosuppression (170). Thus, IFNs display the consequence of resulting in T cell depletion and immunosuppression, although they can also promote the effect of tumor-specific CD8+T cells.

### Immune Contexture

As noted, research of immune checkpoint blockade therapy was concentrated on reversing tumor-specific T cell dysfunction. CD8+T cells play an essential role in the scope of T cell-directed immunotherapy. Thus, the exhaustion of CD8+T cells induced by several factors can also be a crucial reason for PD-1 blockade resistance (143).

Epigenomic modifications might underlie CD8+T cell exhaustion. These long-lasting, exhaustion-associated epigenetic programs limit the rejuvenation of antigen-specific CD8 T cells during PD-1 blockade therapy. A study displayed that initial DNA-methylation programs could restrict T-cell expansion and clonal diversity during PD-1 blockade treatment (171). The administration of DNA-demethylating agents before ICB therapy reversed these programs and enhanced the reinvigoration of antitumor CD8 T cells. Moreover, the latest clinic trials concerning epigenetic therapies also suggest that histone deacetylase inhibitors may synergize with PD-1 blockade to overcome resistance (172, 173). What they found highlights epigenetic programs among exhausted T-cells as a potential mechanism to explain PD-1 blockade therapeutic failures. Besides, research found that co-stimulatory molecules like CD28 can also suppress the function of effector T cells and reduce the response to anti-PD-1 therapy by blocking the CD28-B7 co-stimulatory pathway (13). In addition to the regulation of epigenetic change and co-stimulatory pathway over CD8+T cells, other immune-suppressive cells also have more or less indirect effects on it, impacting drug resistance of anti-PD-1 therapy.

MDSCs are defined as immature myeloid cells, which can be induced to expand by tumor progression and play an immunosuppressive role in multiple cancers (174, 175). The recruitment of immunosuppressive MDSCs has shown complex protumorigenic outcomes following anti-PD-1 therapy (176). One mechanism of this recruitment may be driven by anti-PD-1-activated T cells, which partially trigger a tumor-intrinsic NLRP3 inflammasome signaling cascade (176, 177). This signaling cascade constitutes an adaptive resistance pathway, the genetic and pharmacological inhibition of which can enhance the efficacy of anti-PD-1 immunotherapy by inhibiting the tumor infiltration of MDSCs (176). On the other hand, checkpoint-activated CD8+ T cells can induce the differentiation and survival of protumorigenic TAMs and MDSCs by stimulating tumor production of CSF1 by secreting more TNF-α (178). These prompt us to hypothesize that neutralizing MDSCs and preserving T cell function may elicit robust immunotherapy responses by the combined actions of ICB agents together with targeted agents (179). Paradoxically, in HNSCC, it demonstrates reduced granulocytic MDSC infiltration post-PD-1 blockade (180). Thus, it is still unclear whether this model involves different mechanisms of MDSC recruitment or whether blockade of PD-1 inhibits MDSC proliferation directly.

TAM is another type of myeloid cells. It can impact the response to immunotherapy by activating triggering receptors expressed on myeloid cells 2 (TREM2) (181). TREM2 deficiency was associated with the transformation of macrophage subsets and an increase of intratumoral CD8^+^ T cells, some of which expressed PD-1. The observation found that tumor macrophage infiltrates enhanced T-cell-mediated control of tumor growth after the anti-TREM2 therapy; the anti-TREM2 mAb to tumor-bearing mice blunted tumor growth and strongly enhanced the efficacy of anti-PD-1 immunotherapy (181). Efforts are currently ongoing to complement checkpoint blockade with treatment targeting myeloid cells (115), including depletion of myeloid cells from tumors, blocking their pro-tumoral functions, or restoring their immunostimulatory properties (182, 183). These results may be applied as a theoretical basis to clinical trials.

Tregs can inhibit TCR-mediated activation and proliferation of CD4+/CD8+T cells to promote tumor immune evasion. Simultaneously, EZH2 has a critical role in maintaining the identity and function of Tregs; it has been proved that Ezh2 deficiency in Tregs stimulates antitumor immunity with enhanced T cell infiltration and elevated effector function (147). Mechanistically, Ezh2 functioned in regulating the stability of Foxp3 protein which is specifically expressed by Tregs. Based on these, the synergistic impact of the combination of EZH2 inhibition and anti-PD-1 has been found in an anti-PD-1-resistant model of HNSCC. It is explained that EZH2 inhibition can enhance tumor cell Class I MHC expression *in vivo* including in highly resistant models (147). Thus, it is promising that try to improve the efficacy of anti-PD-1 therapy by combining it with Ezh2 inhibitors.

CAFs are activated fibroblast cells during cancer development, contributing to the establishment of an immunosuppressive TME (184). Despite T cells being recovered from the capability against tumor cells following anti-PD-1 therapy, CAFs can act as a formidable barrier to T cells by secreting-related factors, resulting in T cell exclusion from tumor nests (185). TGF-β, a factor released by CAFs, promotes T-cell exclusion and blocks Th1 effector phenotype acquisition, which eventually results in resistance to PD-1 blockade therapy (186, 187). Inhibition of TGF-β unleashed a potent, enduring cytotoxic T-cell against tumor cells to prevent refractory. In mice with progressive liver metastatic disease, blockade of TGF-β signaling improves the susceptibility to anti-PD-1 therapy and suggests that TGF-β inhibition could prevent, but not reverse, CAF differentiation (186). NOX 4 is a specific downstream target of TGF-β. Inhibition of NOX 4 can “normalize” CAF to a quiescent phenotype and promote intratumoral CD8+ T-cell infiltration, overcoming the exclusion effect (185). These trials show that the regulation of CAFs through repressing the related downstream pathway or factors may have a synergistic effect on the anti-PD-1 therapy.

As mentioned above, one of the major obstacles that remain to be overcome is the restriction of T cells’ function in the immunosuppressive microenvironment formed by Tregs, MDSCs, and TAMs. The adoptive cell therapy (ACT) with chimeric antigen receptor (CAR)-redirected T cells is an attractive anticancer strategy. The breakthrough with CAR-T cell therapy was achieved, targeting B-cell hematologic tumors (188–191), while there is less efficacy in solid tumors. Research shows that TGF-β can be produced in most human tumors and markedly inhibits tumor antigen-specific cellular immunity. CAR-T lymphocytes have generated the resistance to TGF-β suppression, which expresses dominant-negative TGF-β receptors, to counteract these immunomodulatory activities (192). Such a result demonstrates their superior antitumor activity in animal models. Thus, combining engineered CAR-T cells with PD-1 antagonists makes a great deal of sense. There are promising results in both the pre-clinic model and case report (193, 194), presenting a large opportunity for the field of cellular engineering and immune checkpoint therapy.

Accordingly, the abovementioned studies indicate that the resistance to PD-1 inhibitors is directly related to the dysfunction of T cells caused by its epigenetic change, while other immune-related cells can also indirectly result in immune evasion *via* impacting the antitumor immunity progression of T cells.

### Angiogenesis

The angiogenic tumor vasculature plays a vital role in regulating the response to cancer immunotherapy. Vascular abnormalities restrict T cell trafficking into the intratumor *via* upregulating vascular endothelial growth factor (VEGF) and gene-related to proangiogenic (195). Study has suggested that the VEGF signal induces the expression of the factor-related apoptosis antigen ligand (FasL)-mediated cell death on vascular endothelial cells, which in turn poses a formidable physical barrier to vascular material exchange (195). Additionally, the tumor neovasculature also decreases immature DCs and expands Treg cells and MDSC populations (195, 196). The modulation of tumor vasculature includes anti-angiogenesis and vascular normalization, which can induce the depletion of Tregs and regulatory B cells, enhancement of M1 TAMs, and activation of T cells, to reduce immunosuppression. The modulation can make favorable conditions for the infiltration of CD8+ cells and allow the effectiveness of immune checkpoint blockade (197). Immune checkpoint inhibitors have also shown promise in combination with anti-angiogenic in solid tumors (198), such as NSCLC and colorectal cancer (199). Thus, anti-angiogenesis and immunotherapy are documented to work synergistically together, showing promise for the resistance of PD-1 inhibitors.

### Deregulation of Immunometabolism

Immune cells undergo complex shifts in metabolic states; immunosuppressive metabolites in TME can inhibit antitumor immunity by inhibiting immune cell infiltration (200–203).

Aerobic glycolysis is indispensable to CD8+T effector cells. It can be restricted by tumor cells that outcompete T cells for glucose uptake (81). In pretreatment of melanoma tumors, hypoxia-associated genes are highly expressed in the tumors that are subsequently resistant to PD-1 blockade compared with those from responding tumors (204). A high concentration of lactic acid can also blunt aerobic glycolysis of CD8+T cells and correlate with primary resistance on PD-1 blockade (205). A database analysis of patients with melanoma revealed strong negative associations between tumor lactate dehydrogenase expression and markers of CTL activation (201). Separately, indoleamine 2,3-dioxygenase (IDO), generated by tumors and immune cells, can enhance Treg and MDSC production and activity and inhibit the effect on T-cell immunity (206). IDO is the initial and rate-limiting enzyme in the degradation of tryptophan through the kynurenine pathway. A report found a significantly higher kynurenine/tryptophan ratio in NSCLC patients with early progression on nivolumab, suggesting that IDO might contribute to primary resistance to anti-PD-1 monoclonal antibodies (207). Despite that, the following clinical studies have shown that the efficacy of the IDO1 selective inhibitor plus PD-1 inhibitor is not as good as that of PD-1 blockade treatment alone (208). The combination therapy of IDO inhibitors and PD-1 antibodies may become a study direction for overcoming immunotherapy resistance. In addition, adenosine also is an immunosuppressive molecule that can suppress effector T cells and NK cells and increase Treg numbers (209, 210). Accordingly, metabolic disorders can encumber proper T cell activation and effector functions, which is a potential mechanism of resistance to PD-1 blockade. It is believed that the combined strategy based on this can bring gratifying results.

### Disorder of Intestinal Microbiota

The gastrointestinal microbiome has been demonstrated to play an essential role in regulating the immune response function during cancer therapy (211–214). There is a group of active microorganisms that live in symbiosis with the host in the human intestinal tract and may cause tumor resistance to anti-PD-1 when it gets disordered (215, 216). Concordantly, a result has displayed that the responders to PD-1 blockade had a differential composition of gut bacteria (217). It has shown an “unfavorable” gut microbiome with low diversity and high relative abundance. Such a population may impair systemic and antitumor immune responses mediated by the limited intratumoral T cells, myeloid infiltration, and weakened antigen presentation capacity (211). Enhanced responses of anti-PD-1 therapy have been observed in mice that accepted fecal microbiome transplantation of the responder to PD-1 blockade. On the other hand, the efficacy of anti-PD-1 in mice receiving a non-responder could be restored by administration of specific genera enriched in responding patients in these mice. In addition, these specific genera were associated with increased intratumoral immune infiltrates mediated by the recruitment of CD4^+^T cells into the tumor bed and increased ratio of CD4^+^T cells to Tregs in response to PD-1 blockade (217). Besides, fecal microbiota transplant also overcomes resistance to anti-PD-1 therapy in melanoma patients (218). This suggests that regulating the gut microbiota may potentially enhance antitumor immune responses as well as response to immune checkpoint blockade.

### New Immune Checkpoints

During checkpoint blockade with anti-PD-1 inhibitors, other inhibitory checkpoints might become coordinately upregulated and in turn lead to therapeutic failure (219). T-cell immunoglobulin mucin 3 (TIM-3), a member of the TIM family of immunomodulatory proteins, has been identified as a critical regulator of CTL exhaustion with co-expression of PD-1 (220). Such co-expression means that the most dysfunctional subgroup of T cells does not produce IL-2 and IFN-γ and eventually causes adaptive resistance. The mechanism has demonstrated that the increased Tim-3-mediated escape of exhausted TIL from PD-1 inhibition was mediated by PI3K/Akt complex downstream of TCR signaling in HNSCC (219). *In vitro*, the anti-Tim-3-blocking antibody reverses resistance to anti-PD-1 in PBMC from lung cancer patients (221). On the other hand, significant antitumor activity was observed after sequential addition of anti-Tim-3 mAb to overcome adaptive resistance to anti-PD-1 mAb in a murine HNSCC model (219). Thus, combination therapy targeting TIM-3 and PD-1 signaling pathways might be effective against the resistance of mono-immunotherapy.

Lymphocyte activation gene 3 (LAG-3) can selectively be expressed on activated T cells, NK cells, DCs and may get compensatory upregulation. The regulatory function of LAG-3 on T cells is similar to that of PD-1, which delivers suppressive signaling to hinder antitumor response (222). LAG-3 also competes for binding to MHC class II, which leads to decreased efficacy of MHC class II-mediated antigen presentation (223). The upregulation of LAG-3 in tumors of melanoma and lung cancer patients with acquired resistance to anti-PD-1 therapy has been demonstrated (223). There appeared to be a synergistic benefit of anti-LAG-3/anti-PD-1 combinatorial immunotherapy compared with anti-PD-1 monotherapy. In addition, a higher proportion of effector T cells were observed in mice treated with anti-LAG-3/anti-PD-1 than in PD-1 monotherapy groups. These suggest that anti-LAG-3/anti-PD-1 combinatorial immunotherapy may act synergistically (224). The roles of other checkpoints are still unconfirmed in anti-PD-1 resistance, such as TIGIT. Thus, a more particular knowledge of these new immune checkpoints may provide a rationale for designing combination treatments in the future.

## Conclusions

In this review, we primarily describe a complex story of the relationship between anti-PD-1 and TME. The initiation of the antitumor effect depends on the cross talk between immune cells (**Figure 1**). Besides T cells, other immune-activating cells, like NK cells, DCs, and M1 TAMs, also contribute to anti-PD-1 efficacy through direct or indirect mechanisms. Furthermore, PD-1 blockade can target PD-1 expressed on these cells directly or reactivate CD8+ T cells to induce these immune-activating cell responses indirectly within the TME. Also, the review briefly displays the mechanisms that possibly contribute to primary or acquired resistance to PD-1 blockade, including the internal and external causes; the former focuses on the inherent characteristics of tumor cells while the other is mainly emanated from the tumor microenvironment (**Figure 2**). Due to the different reasons for drug resistance, the appropriate combination immunotherapy is also different, which is also discussed in detail in this article. It means that using a combination of such strategies is more suitable than using one approach alone for stimulating an antitumor immune response in some situations. A future challenge for researchers and clinicians is to achieve the satisfactory efficacy of immunotherapy. It means that the mechanisms of tumor immune evasion and immune drug resistance should be clarified as much as possible. It also plays a crucial role in the exploration of predictive markers, which are associated with the response rate of immunotherapy and improved clinical outcomes.

## Author Contributions

QW wrote the manuscript; all authors were involved in the amendments and improvements in the text. All authors contributed to the article and approved the submitted version.

## Funding

This work was supported by the National Natural Science Foundation of China [82072594 (YT), 82073097 (SL), 82073136 (DX), 81874139 (SL), and 81872285 (YS)], Shenzhen Science and Technology Program [KQTD20170810160226082 (YT)], Shenzhen Municipal Government of China [JCYJ20180507184647104 (YT)], and the Hunan Provincial Key Area R&D Program [2019SK2253 (YT)].

## Conflict of Interest

The authors declare that the research was conducted in the absence of any commercial or financial relationships that could be construed as a potential conflict of interest.

## Publisher’s Note

All claims expressed in this article are solely those of the authors and do not necessarily represent those of their affiliated organizations, or those of the publisher, the editors and the reviewers. Any product that may be evaluated in this article, or claim that may be made by its manufacturer, is not guaranteed or endorsed by the publisher.

## Glossary

**Table:**

APC	antigen-presenting cell
ARG1	Arginase 1
B2M	β2-microglobulin
cHL	classical Hodgkin’s lymphoma
CTL	cytotoxic T lymphocyte
CAF	cancer-associated fibroblast
DC	dendritic cell
DLBCL	diffuse large B cell lymphoma
DNMTi	DNA methyltransferase inhibitor
EZH2	enhancer of zeste homolog 2
FDA	Food and Drug Administration
FasL	factor-related apoptosis antigen ligand
FL	follicular lymphoma
HNC	head and neck cancer
HNSCC	head and neck squamous cell carcinoma
H3K27me3	histone h3 lysine 27 tri-methylation
ICB	immune checkpoint blocker
ITIM	immunoreceptor tyrosine-based inhibitory motif
ITSM	immunoreceptor tyrosine-based switch motif
ILC	innate lymphoid cell
IDO	indoleamine 2,3-dioxygenase
IFN	interferon
iNOS	inducible nitric oxide synthase
JAK	Janus kinase
LSD1	lysine-specific histone demethylase 1
LAG-3	lymphocyte activation gene 3
MHC	major histocompatibility complex
MAPK	mitogen-activated protein kinase
mCRPC	metastatic castration-resistant prostate cancer
mAb	monoclonal antibody
MDSC	myeloid-derived suppressor cell
NK	natural killer cell
NSCLC	non-small cell lung cancer
NCI	National Cancer Institute
NF-κB	nuclear factor kappa-light-chain-enhancer of activated B cells
PD-1	programmed cell death receptor 1
PD-L1	programmed cell death ligand 1
PD-L2	programmed cell death ligand 2
PRC2	polycomb repressive complex 2
PTEN	phosphatase and tensin homolog
RT	radiotherapy
STAT1	signal transducer and activator of transcription 1
TDLNs	tumor-draining lymph nodes
TME	tumor microenvironment
TCR	T cell receptor
TAM	tumor-associated macrophage
TNF-α	tumor necrosis factor α
Th1 cell	T helper 1 cell
Treg	regulatory T cell
TGF-β	transforming growth factor β
TREM2	triggering receptor expressed on myeloid cells 2
TIM-3	T-cell immunoglobulin mucin 3
UC	urothelial carcinoma
VEGF	vascular endothelial growth factor
